# Supplementary material for: Structural and Photophysical Properties of 2,1,3-Benzothiadiazole-Based Phosph(III)azane and Its Complexes
Source: Molecules. 2020 May 22;25(10):2428. doi: 10.3390/molecules25102428 (PMC7288126; doi:10.3390/molecules25102428)
Supplement: Supplementary file 1 [file molecules-25-02428-s001.pdf]

*molecules*

*Supplementary information*

# Structural and Photophysical Properties of 2,1,3-Benzothiadiazole-Based Phosph(III)azane and Its Complexes

**Radmir Khisamov, Taisiya Sukhikh, Denis Bashirov, Alexey Ryadun and Sergey Konchenko**

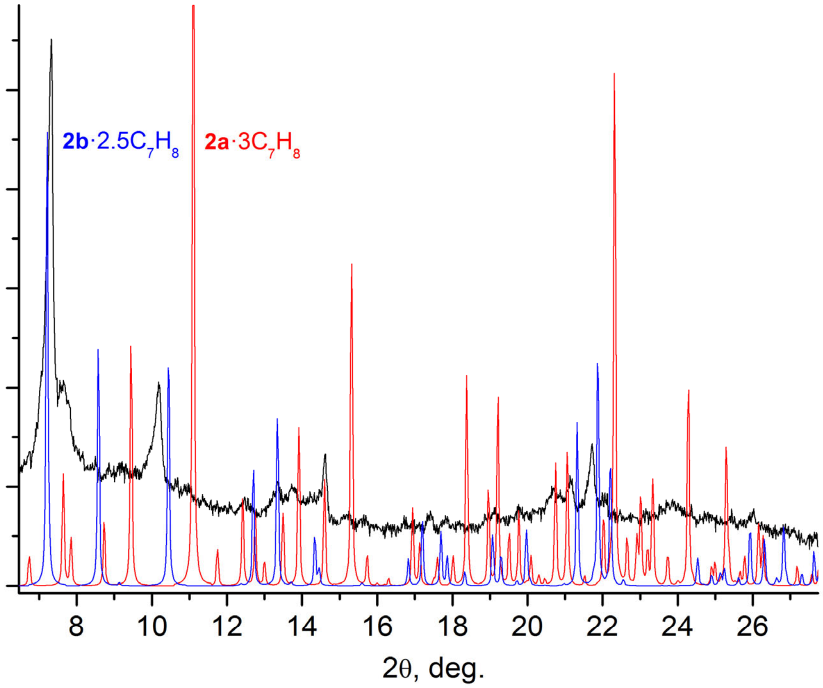


**Figure S1.** Experimental (black) and simulated (red and blue) powder patterns of the compounds **2a**·$3C_7H_8$ and **2b**·$2.5C_7H_8$.

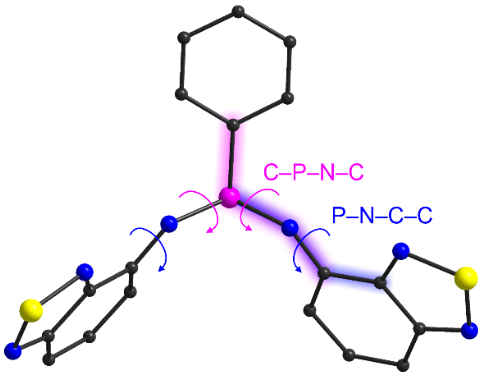


**Figure S2.** Representation of selected torsion angles in $H_2L$ or $L^{2-}$ fragments. Hydrogens are not shown.

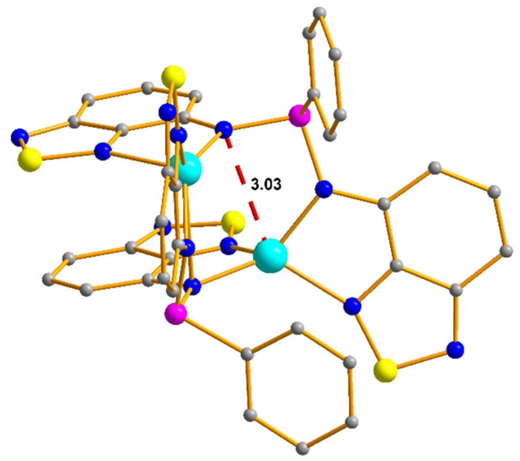


**Figure S3.** Molecular structure of **1** showing attraction interaction Zn–N[4'] marked by dashed red line.

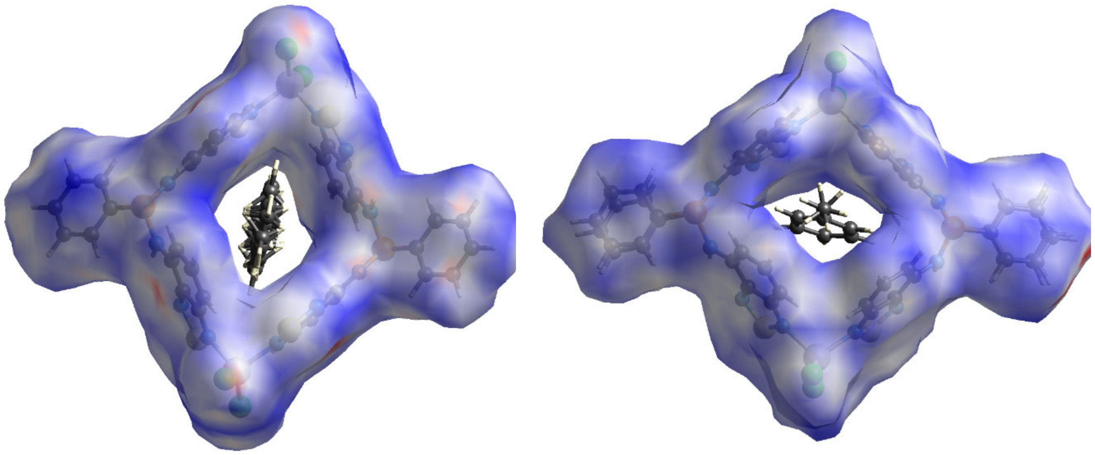

**Figure S4.** The $d_{norm}$ Hirshfeld surface of the complexes in crystal structures **2a**·3$C_7H_8$ (left) and **2b**·2.5$C_7H_8$ (right). Area with intermolecular contacts closer than the sum of atoms van der Waals radii are red, longer contacts are blue, and the contacts around the sum of van der Waals radii are white.

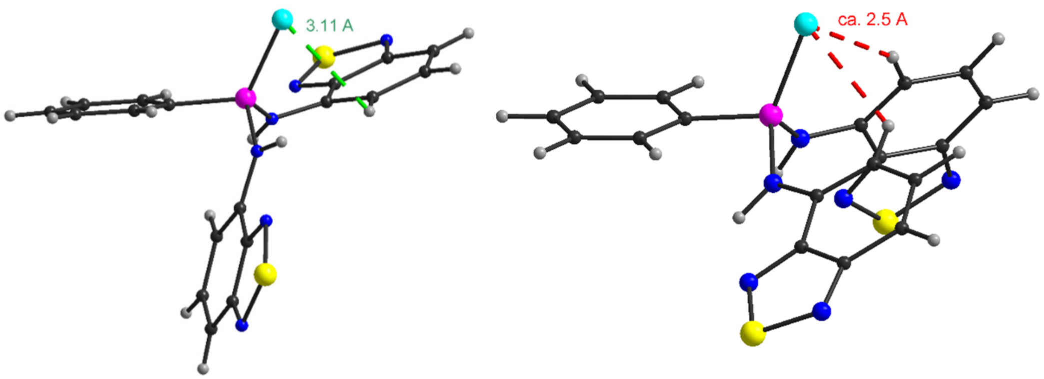

**Figure S5.** Representation of a Cu–$H_2L$ model with the geometry derived from XRD data for free $H_2L$ (left; Cu atom was placed geometrically) and the corresponding fragment from XRD data for **3** (right). Red and Green dashed lines indicate Cu···H repulsion interactions.

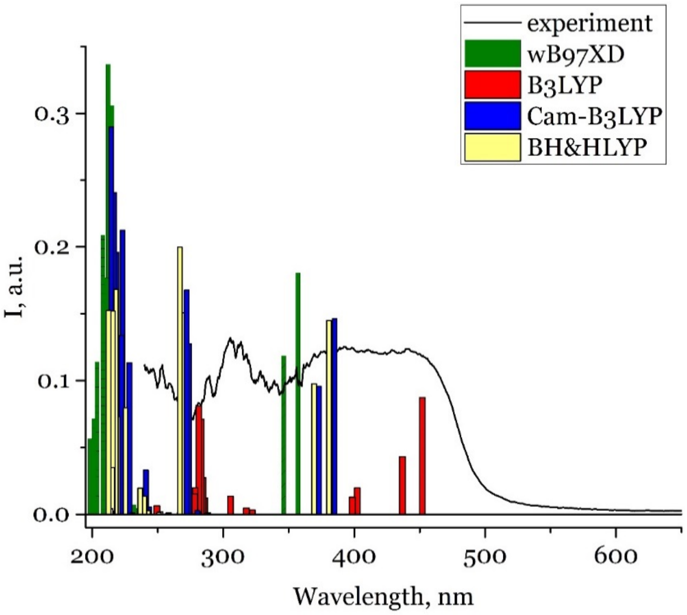


**Figure 6.** Overlaid TD-DFT calculations results and experimental UV-Vis spectrum of $H_2L$.

**Table S1.** Calculated properties of the first singlet excited states $S_0 \rightarrow S_n$ of $H_2L$: transition wavelength ($\lambda$), oscillator strength (f). H is for HOMO, L is for LUMO abbreviation.

| n | λ, nm | f | electronic states | contribution |
|---|---|---|---|---|
| 1 | 452.1 | 0.0872 | H→L | 0.9438 |
| 2 | 436.8 | 0.0431 | H→L+1 | 0.9520 |
| 3 | 402.3 | 0.0196 | H-1→L | 0.9563 |
| 4 | 398.5 | 0.0128 | H-1→L+1 | 0.9460 |
| 5 | 322.3 | 0.0030 | H-2→L | 0.9845 |
| 6 | 317.6 | 0.0045 | H-2→L+1 | 0.9818 |
| 7 | 305.6 | 0.0135 | H→L+2 | 0.9825 |

**Table S2.** Crystal data and structure refinement for $H_2L$, **1–4.**

| **Identification code** | **$H_2L$** | **1** | **2a·3$C_7H_8$** | **2a·2.5$C_7H_8$** | **3·THF** | **4** |
|---|---|---|---|---|---|---|
| Empirical formula | $C_{18}H_{13}N_6PS_2$ | $C_{50}H_{38}N_{12}P_2S_4Zn_2$ | $C_{57}H_{50}Cl_4N_{12}P_2S_4Zn_2$ | $C_{53.5}H_{46}Cl_4N_{12}P_2S_4Zn_2$ | $C_{22}H_{21}ClCuN_6OPS_2$ | $C_{30}H_{19}N_9P_2S_3$ |
| Formula weight | 408.43 | 1127.84 | 1365.81 | 1319.74 | 579.53 | 663.66 |
| Space group | $Pna2_1$ | $C2/c$ | $C2/m$ | $P4_22_12$ | $C2/c$ | $P$–1 |
| a/Å | 17.8032(12) | 19.7209(12) | 14.349(3) | 15.9225(8) | 23.7977(11) | 10.119(4) |
| b/Å | 22.9545(17) | 19.3027(11) | 24.505(4) | 15.9225(8) | 15.1196(9) | 11.337(4) |
| c/Å | 4.3288(3) | 13.0051(8) | 10.6832(18) | 23.1212(14) | 16.4906(9) | 13.556(5) |
| α/° | 90 | 90 | 90 | 90 | 90 | 81.340(12) |
| β/° | 90 | 103.554(2) | 127.670(5) | 90 | 123.116(2) | 73.235(12) |
| γ/° | 90 | 90 | 90 | 90 | 90 | 72.680(13) |
| Volume/Å$^3$ | 1769.0(2) | 4812.7(5) | 2973.4(9) | 5861.8(7) | 4969.7(5) | 1418.0(10) |
| Z | 4 | 4 | 2 | 4 | 8 | 2 |
| $\rho_{calc}$g/cm$^3$ | 1.534 | 1.557 | 1.526 | 1.495 | 1.549 | 1.554 |
| μ/mm$^{-1}$ | 0.409 | 1.288 | 1.231 | 1.246 | 1.247 | 0.416 |
| F(000) | 840.0 | 2304.0 | 1396.0 | 2692.0 | 2368.0 | 680.0 |
| Crystal size/mm$^3$ | 0.18 × 0.14 × 0.06 | 0.14 × 0.14 × 0.08 | 0.15 × 0.12 × 0.1 | 0.12 × 0.09 × 0.09 | 0.22 × 0.14 × 0.1 | 0.08 × 0.06 × 0.03 |
| 2Θ range for data collection/° | 2.894 to 46.488 | 4.018 to 48.924 | 3.952 to 48.808 | 3.106 to 49.068 | 3.68 to 55.95 | 3.146 to 49.1 |
| Index ranges | -16 ≤ h ≤ 19, -21 ≤ k ≤ 25,-4 ≤ l ≤ 3 | -22 ≤ h ≤ 19, -22 ≤ k ≤ 21,-14 ≤ l ≤ 15 | -16 ≤ h ≤ 16, -28 ≤ k ≤ 25,-11 ≤ l ≤ 12 | -18 ≤ h ≤ 18, -18 ≤ k ≤ 18,-26 ≤ l ≤ 22 | -31 ≤ h ≤ 30, -19 ≤ k ≤ 19,-21 ≤ l ≤ 21 | -11 ≤ h ≤ 11, -13 ≤ k ≤ 13, -14 ≤ l ≤ 15 |
| Reflections collected | 4262 | 11184 | 7651 | 63517 | 17435 | 15306 |
| Independent reflections | 2043 [$R_{int}$ = 0.0755, $R_{sigma}$ = 0.0806] | 3952 [$R_{int}$ = 0.0310, $R_{sigma}$ = 0.0433] | 2515 [$R_{int}$ = 0.0549, $R_{sigma}$ = 0.0742] | 4898 [$R_{int}$ = 0.0723, $R_{sigma}$ = 0.0357] | 5963 [$R_{int}$ = 0.0285, $R_{sigma}$ = 0.0362] | 4672 [$R_{int}$ = 0.1012, $R_{sigma}$ = 0.1073] |
| Restraints/parameters | 3/251 | 0/317 | 43/168 | 254/396 | 2/314 | 96/397 |
| Goodness-of-fit on F$^2$ | 1.029 | 1.028 | 1.007 | 1.029 | 1.038 | 1.034 |
| Final R indexes [I>=2σ (I)] | $R_1$ = 0.0446, w$R_2$ = 0.0956 | $R_1$ = 0.0338, w$R_2$ = 0.0794 | $R_1$ = 0.0495, w$R_2$ = 0.1182 | $R_1$ = 0.0338, w$R_2$ = 0.0770 | $R_1$ = 0.0334, w$R_2$ = 0.0777 | $R_1$ = 0.0887, w$R_2$ = 0.2166 |
| Final R indexes [all data] | $R_1$ = 0.0649, w$R_2$ = 0.1042 | $R_1$ = 0.0485, w$R_2$ = 0.0860 | $R_1$ = 0.0830, w$R_2$ = 0.1320 | $R_1$ = 0.0465, w$R_2$ = 0.0825 | $R_1$ = 0.0449, w$R_2$ = 0.0829 | $R_1$ = 0.1238, w$R_2$ = 0.2399 |

| | | | | | | |
|---|---|---|---|---|---|---|
| Largest diff. peak/hole / e $Å^{-3}$ | 0.36/-0.31 | 0.58/-0.41 | 0.81/-0.89 | 0.60/-0.48 | 0.43/-0.36 | 0.87/-0.79 |
| Flack parameter | 0.39(18) | | | 0.009(7) | | |
